# Differential regulation of vaginal lipocalins (OBP, MUP) during the estrous cycle of the house mouse

**DOI:** 10.1038/s41598-017-12021-2

**Published:** 2017-09-15

**Authors:** Martina Černá, Barbora Kuntová, Pavel Talacko, Romana Stopková, Pavel Stopka

**Affiliations:** BIOCEV group, Department of Zoology, Faculty of Science, Charles University, Viničná 7, Prague, CZ 12844 Czech Republic

## Abstract

Female house mice produce pheromone-carrying major urinary proteins (MUPs) in a cycling manner, thus reaching the maximum urinary production just before ovulation. This is thought to occur to advertise the time of ovulation via deposited urine marks. This study aimed to characterize the protein content from the house mouse vaginal flushes to detect putative vaginal-advertising molecules for a direct identification of reproductive states. Here we show that the mouse vaginal discharge contains lipocalins including those from the odorant binding (OBP) and major urinary (MUP) protein families. OBPs were highly expressed but only slightly varied throughout the cycle, whilst several MUPs were differentially abundant. MUP20 or ‘darcin’, was thought to be expressed only by males. However, in females it was significantly up-regulated during estrus similarly as the recently duplicated central/group-B MUPs (sMUP17 and highly expressed sMUP9), which in the mouse urine are male biased. MUPs rise between proestrus and estrus, remain steady throughout metestrus, and are co-expressed with antimicrobial proteins. Thus, we suggest that MUPs and potentially also OBPs are important components of female vaginal advertising of the house mouse.

## Introduction

During an estrous cycle, most mammalian females will pass through the four consecutive phases including proestrus, estrus, metestrus, and diestrus (e.g. *Mus* ^1, 2^, *Apodemus* ^3^, *Rattus* ^4^). The estrous cycle in mice is strongly affected by pheromones. Suppression of estrus in single-sex populations^5^, rapid return to estrus when paired with males^6^ or exposed to male urine^7^ are well known effects and they were also observed in other mammals^3, 8^. The house mouse (*Mus musculus*) uses a system of lipocalin transporters of active volatile organic compounds–VOCs^9–12^, which together serve as signals. In mice, these signals are manifested via expression of large quantities of male-biased^13^ and highly homologous^14–16^ major urinary proteins (MUPs) in the liver. MUPs are products of a gene cluster on the chromosome 4, that contains ~21 coding genes (and a similar number of pseudogenes), and can be divided into two groups, the group-A (ancestral), containing *Mup3*, *Mup4*, *Mup5*, *Mup6*, *Mup20* and *Mup21*, and the group-B, consisting of 15 other *Mup*s (i.e. *Mup1*, *Mup2*, *Mup7-Mup19*) sharing almost 99% sequence identity^17, 18^, reviewed in citations^19, 20^. They bind some volatile organic compounds (VOCs) in their eight-stranded beta barrel and transport them to the outside environment with urine^10, 21–23^, where they act as an honest, cheat-proof display of an individual’s health and condition^24^. Such scent-marking signals have strong effects on the reproductive success of the signaller^25^. Interestingly, group-A and group-B MUPs are not just in the urine, they were recently identified also in tears^26^ and saliva^27^. However, most studies focused on the male components of chemical signalling and, thus, there is very little evidence on the roles of female MUPs.

A group of lipocalins that may also be involved in the transport of chemical signals and potentially in the mouse vaginal advertising are members of the odorant binding protein family (OBPs), because in other mammals, they were detected in their vaginal secretions (e.g. hamsters^28^ and potentially bank voles^29^) and/or urine (e.g. mole rats^30^ and bank voles^29^). The X-linked *Obp* genes of the mouse were thought to involve just the two nasal members – *Obp1a*, and *Obp1b* ^31^. However, *Obp* genes have also undergone a series of duplications in mice, and they occur in a cluster of seven genes (i.e. including *Prb*, Probasin) and two pseudogenes on the X chromosome^32, 33^. In wild *M*. *m*. *musculus* mice, *Obp*s were predicted from the genome data^19^, detected as transcripts (*Obp1* - KJ605390, *Obp2 -* KJ605391, *Obp5* (*Obp1a*) - KJ605392, *Obp6* - KJ605393, and *Obp7* (*Obp1b*) - KJ605394)^33^ and their expression was corroborated on the level of protein in the mouse tears^26^ and saliva^27^. Mouse OBPs are produced by various oro-facial tissues including olfactory (OE), vomeronasal (VNO), nasal-lymphoid, and lacrimal glands/tissues^26, 33^ and they are naturally transported with mucosal secretions to the oral cavity, where they are highly abundant^27^. It has also been demonstrated that they are involved in the process of rapid ligand internalization by means of removing small organic molecules including odorants from nasal mucosa, this has been demonstrated for OBP5 (i.e. OBP1a in olfactory epithelia)^34^. However, data on these proteins from the mouse vaginal secretions were missing.

In mice, chemical signalling was thought to be manifested via urine marks, and so many experiments have been performed to study in detail this component of chemical signalling. For example, female house mice use major urinary proteins to advertise their estrus with urine marks. It has been shown in the laboratory mice^35^ and in wild house mice (*M*. *m*. *musculus*)^36^ that (i) males up-regulate urinary MUPs during social contacts, and that (ii) females use MUPs to advertise their reproductive states by varying the concentration of MUPs in the urine during the estrous cycle^35^. The urine volatiles during estrus are recognized by a synergistic action of the VNO-OE system of the mouse, and it has been demonstrated that the main olfactory system (MOS) is involved primarily in the attraction from a distance, while the VNO plays a major role in close proximity during pre-copulatory behaviour^37^. Moreover, there is also a study on rats (*Rattus rattus*) also showing that a lipocalin with chemosignalling functions was highly expressed during estrus and metestrus phases in the urine^38^. Thus, it is possible that the close proximity and chemical signals on bodies of interacting individuals could also be important during sexual interactions because this component of signalling is directly linked to an interacting individual, i.e. the signal owner. Thus, the aim of our study was to analyse the relative protein content of vaginal flushes and its variation throughout the cycle to detect potential signalling proteins, and to evaluate the role of particular estrous phases in regulating the expression of these proteins. Furthermore, we also aimed to determine the level of correlation between the house mouse estrous cycle and the host regulation and degradation of bacteria to further the understanding of the emergence of complex odour mixtures.

## Materials and Methods

### Ethical Standards

All animal procedures were carried out in strict accordance with the law of the Czech Republic paragraph 17 no. 246/1992 and the local ethics committee of the Faculty of Science, Charles University in Prague chaired by Dr. Stanislav Vybíral specifically approved this study in accordance with accreditation no. 27335/2013–17214 valid until 2019.

### Subjects, housing conditions and experimental design

In this experiment, we used a total of 9 G1 wild-derived *Mus musculus musculus* females (120 days old) and 9 unfamiliar males (120 days old) for a period of social stimulation during which vaginal fluid samples were collected on a daily basis. We opted for biological replicates instead of methodological duplicates simply for the fact that in similar studies the duplicates were highly correlated^26^. Females were housed in pairs with a male and in cages divided by a wire mesh from individual males with square openings (diameter was 1 cm) allowing communication but suppressing direct contact (13:11 hrs, D:N, temperature t = 23 °C). The experimental cages were supplied with fresh bedding at the beginning of the experiment and provided with water and food ad libitum. Vaginal lavage was performed daily between 8:00 and 9:00am by gentle flushing with a pipette using 20 μl of the 0.9% saline solution. Samples were centrifuged at 300 rcf for 10 minutes, 4 °C. Supernatant was used for the protein analysis and the cell pellet was used to prepare slides on a concentrator (StatSpin) for further cytological analyses. These slides were differentially stained to visualize nucleated and cornified cells with May-Gruenwald (3 min) and Giemsa (10 min) staining solutions.

### Protein Digestion

Prior to our experiments, we tested the efficiency of ethanol and acetone precipitation of proteins from the vaginal lavage samples. Some acetone precipitated samples did not enter the gel (PAGE) whilst all proteins entered the gel after cold ethanol precipitation. Thus, all protein samples were precipitated with the ice-cold ethanol (20 minutes) and centrifuged at 10 000 rcf for 10 minutes, 0 °C. This was followed by a re-suspension of dried pellets in the digestion buffer (1% SDC, 100 mM TEAB – pH = 8.5). Protein concentration of each lysate was determined using the BCA assay kit (Fisher Scientific). Cysteines in 20 μg of proteins were reduced with a final concentration of 5 mM TCEP (60 °C for 60 min) and blocked with10mM MMTS (i.e. S-methyl methanethiosulfonate, 10 min Room Temperature). Samples were cleaved with trypsin (1 ug of trypsin per sample) in 37 °C overnight. Peptides were desalted on a Michrom C18 column.

### nLC-MS^2^ Analysis

Nano Reversed phase columns were used (EASY-Spray column, 50 cm × 75 µm ID, PepMap C18, 2 µm particles, 100 Å pore size**)**. Mobile phase buffer A was composed of water, and 0.1% formic acid. Mobile phase B contained acetonitrile, and 0.1% formic acid. Samples were loaded onto a trap column (Acclaim PepMap300, C18, 5 µm, 300 Å Wide Pore, 300 µm × 5 mm, 5 Cartridges) for 4 min at 15 μl/min loading buffer was composed of water, 2% acetonitrile and 0.1% trifluoroacetic acid. After 4 minutes ventile was switched and Mobile phase B increased from 4% to 35% B at 60 min, 75% B at 61 min, hold for 8 minutes, and 4% B at 70 min, hold for 15 minutes until the end of run.

Eluting peptide cations were converted to gas-phase ions by electrospray ionization and analysed on a Thermo Orbitrap Fusion (Q-OT-qIT, Thermo). Survey scans of peptide precursors from 350 to 1450 *m*/*z* were performed at 120 K resolution (at 200 *m*/*z*) with a 5 × 10^5^ ion count target. Tandem MS was performed by isolation at 1.5 Th with the quadrupole, HCD fragmentation with normalized collision energy of 30 and rapid scan MS analysis in the ion trap. The MS^2^ ion count target was set to 10^4^ and the max injection time was 35 ms. Only those precursors with charge state 2–6 were sampled for MS^2^. The dynamic exclusion duration was set to 45 s with a 10 ppm tolerance around the selected precursor and its isotopes. Monoisotopic precursor selection was turned on. The instrument was run in top speed mode with 2 s cycles.

### Protein analysis

LC-MS data were analysed and quantified with MaxQuant software (version 1.5.3.8)^39^. The false discovery rate (FDR) was set to 1% for both proteins and peptides and we specified a minimum peptide length of seven amino acids. The Andromeda search engine was used for the MS/MS spectra search against our modified Uniprot *Mus musculus* database (downloaded on June, 2015), containing 44,900 entries. We modified our databases such that all MUP, OBP sequences were removed and instead of them we have added a complete list of MUPs from Ensembl database, and OBPs from NCBI (sensu - citation^33^). Next we added some Tremble sequences that were missing in Uniprot, for example KLKs, BPIs, SPINKs, SCGB/ABPs, and LCNs. Enzyme specificity was set as C-terminal to Arg and Lys, also allowing cleavage at proline bonds^40^ and a maximum of two missed cleavages. Dithiomethylation of cysteine was selected as fixed modification and N-terminal protein acetylation and methionine oxidation as variable modifications. The “match between runs” feature of MaxQuant was used to transfer identifications to other LC-MS/MS runs based on their masses and retention time (maximum deviation 0.7 min) and this was also used in all quantification experiments. Quantifications were performed with the label-free algorithms described recently^39^ using a combination of unique and razor peptides. All statistical analyses were performed in R software^41^. First, the dataset was normalized to diminish potential differences due to differential protein extractability and also due to potential differences caused by different signal intensity between samples. We used a normalization based upon quantiles, which normalizes a matrix of peak areas/intensities with the function normalize.quantiles from ‘preprocessCore’ routines under the Bioconductor package^42^. This method is based upon the concept of a quantile-quantile plot extended to n dimensions. To check that the data distribution conforms to the same type of distribution after normalization, we used ‘mixtools’^43^. Second, we used the Power Law Global Error Model (PLGEM)^44^ to detect differentially expressed/abundant proteins using the functions plgem.fit and plgem-stn^43^. Original and normalized LC-MS/MS data are provided in Dataset 1.

### Data validation with targeted quantification of selected proteins

To corroborate evidence on the variation of protein abundances obtained by LC-MS approach, we have selected several ‘key’ proteins (i.e. significant lipocalins that were discussed in the manuscript) and quantified them by Selected Reaction Monitoring (SRM) using triple quadrupole MS/MS instrument TSQ Quantiva (Thermo Scientific). Targeted SRM assays are the mass spectrometry equivalent of a Western blot and are intended to complement discovery-based analysis^45^. Based on the data from untargeted LC-MS experiment we chose 2 proteotypic peptides per protein for SRM experiment. For each peptide 3 transitions were selected (Dataset 1). LC separation was done with the same setup and conditions as for untargeted LC-MS experiment. Samples were analysed in positive mode with resolution for both Q1 and Q3 set to 0.7, collision gas pressure was set to 1.5 mTorr, cycle time was 1.5 s. Collision energy for each peptide was predicted and results were obtained using Skyline Daily 3.6.1.^46^. Datasets were normalized the same way as LC-MS data – see above.

## Results

### The vaginal fluid proteome of the house mouse

**Figure 1:**
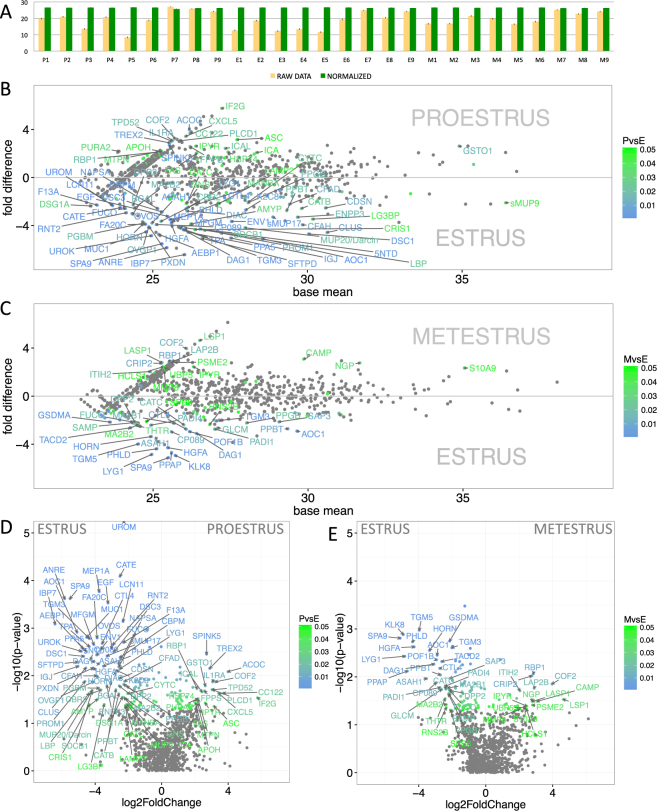
Graphical representation of differentially expressed/abundant proteins. Before normalization, the data revealed some variation between individuals (**A**–yellow bars). However, after the quantile-normalization procedure (**A**–green bars), the mean value and standard error bars show almost no variation between the samples. Significant differentially-expressed proteins are demonstrated with MA plots and are more common in estrus than in proestrus or metestrus (**B**,**C**). PLGEM model was involved in testing the differences in normalized signal values between proestrus and estrus (**B**), and between metestrus and estrus (**C**). The level of significance (PvsE, MvsE) is scaled from green (P < 0.05) to blue (P < 0.01) and only the data points with FC > 2 are annotated. The x-axis represent the basal mean of signal intensities in **B** and **C**. The dependence of particular fold changes on p-values is provided using the volcano plots in **D** and **E**.

We have generated the vaginal fluid proteome of the house mouse, *M*. *m*. *musculus* from 9 females, each in three estrous phases (i.e. proestrus, estrus, metestrus) and detected a total of 2507 proteins at 0.01 FDR (i.e. False Discovery Rate for all peptides and proteins). However, some proteins were detected only in one or two instances per individual. Thus, we reduced our dataset such that all the rows that had just two counts per row were deleted as well as those rows where the median expression in all three groups was lower than 1 (i.e. number of proteins decreased to N = 986). To reduce the influence of false positive abundances due to differences in signal intensities between individuals we quantile-normalized a matrix of protein abundances with ‘preprocessCore’ routine within the *Bioconductor package* in R software^43^; this step ensures that differential expression (i.e. abundances or ‘expression patterns’) is measured instead of differential extractability of proteins from complex mucosal secretions. This normalization strategy has resulted in highly similar datasets with similar data distribution, almost the same mean values, and lower standard deviation (i.e. RAW DATA: 19.2 ± 4.9, NORMALIZED DATA: 26.4 ± 0.1, see Fig. 1A) thus decreasing the potential of obtaining false positive values.

After normalization of our data, many of those proteins that were invariant throughout the cycle and most expressed belong to a group of “housekeeping” genes (HK) which are used in many experiments for normalization. In our data, these included: beta actin (ACTB), serum albumin (ALB), phosphoglycerate kinase 1 (PGK1), fructose-bisphosphate aldolase (ALDOA), Glyceraldehyde 3-phosphate dehydrogenase (G3P, gapdh), and for example all of the 16 detected proteasomal subunits that are provided in Dataset 1. Thus, the lack of any variation in these highly expressed proteins signifies that the quantile-normalization of our data was sufficiently robust and does not require further HK normalization.

Next, we searched for differentially expressed proteins throughout the cycle using the Power Law Global Error Model (PLGEM)^44^. This model was first developed to quantify microarray data^44^, however, due to similar statistical properties – namely the n-binomial distributions of signal values (i.e. deviating from normality) – it has proved to be an amenable model for the quantification of label-free MS-based proteomics data^47^. We calculated the signal-to-noise ratio – STN (equation provided in citation^47^), because it explicitly takes unequal variances into account and because it penalizes proteins that have higher variance in each class more than those proteins that have a high variance in one class and a low variance in another^44^. To create statistical baseline, PLGEM can only be fitted on a set of replicates from the same experimental condition, so we have done this for estrus phase. Correlation between the mean values and standard deviations was high (r^2^ = 0.985, Pearson = 0.977, Supplementary Fig. 1) so we continued with the resampled STNs and calculated differences with corresponding p-values between proestrus and estrus, and between metestrus and estrus. Mean value differences between proestrus and estrus (Fig. 1B), and metestrus and estrus (Fig. 1C) are visualized with MA plots (only protein names with P < 0.05 and fold change FC > 2 or FC < −2 are shown), where in both the plots Fig. 1B,C it is obvious that prevailing number of proteins that are differentially expressed are those that are abundant in estrus (i.e. lower parts of Fig. 1B,C and left parts of the volcano plots in Fig. 1D,E). The plot also demonstrates that the most expressed and at the same time differentially regulated protein is sMUP9 (proestrus→estrus 2 fold, see below).

### Differentially expressed proteins – markers of estrus

**Figure 2:**
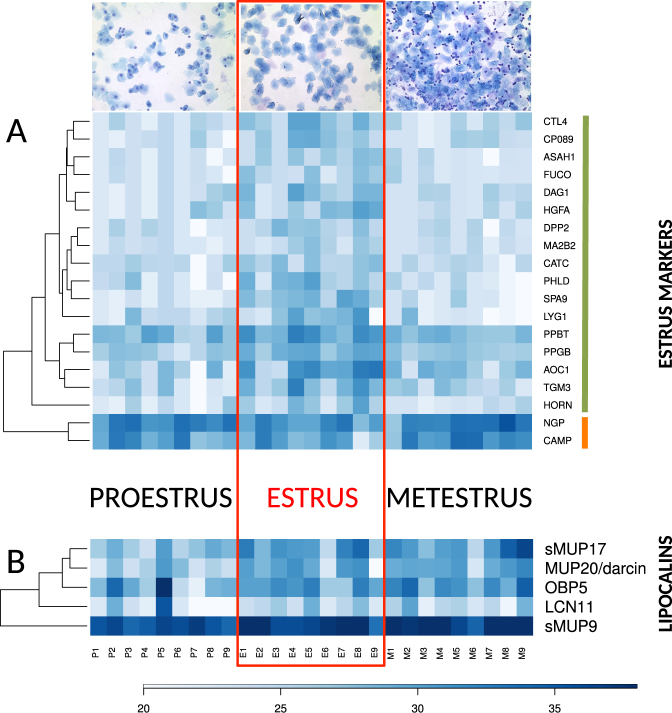
Graphical representation of individual changes in protein abundances (FC > 2 and P < 0.05) with heat maps. (**A**)–shows significant proteins abundant only in estrus (i.e. estrus markers, green bar next to the gene symbols) and the two bactericidal proteins NGP and CAMP which were downregulated in estrus (orange bar), these two groups were reliably separated by a hierarchical clustering method, (**B**)–shows the significant lipocalins MUP20-darcin, sMUP9, and sMUP17 with a notable variation between individuals. We have also added OBP5 with FC < 2 (i.e. 1.6 fold, P = 0.0027). Representative microphotographs of the vaginal cytology were taken at magnification 100x after May-Gruendewald and Giemsa staining.

PLGEM analysis of protein abundances on the level of FC > 2 revealed that a total of 69 proteins (i.e. 7%) were up-regulated in estrus. Of these 69 proteins, a total of 17 proteins (1.7%) were consequently down-regulated in metestrus (Fig. 2A), whilst 52 (5.3%) of them remained unchanged on the level of P < 0.05 and FC > 2. This means that only 1.7% of all proteins were detected as significant markers of estrus. These are: SPA9, LYG1, PHLD, HGFA, ASAH1, HORN, DAG1, CP089, AOC1, CTL4, PPBT, CATC, MA2B2, PPGB, DPP2, FUCO, TGM3. Gene ontology analysis with STRING (https://string-db.org/) revealed that – on the level of FDR = 0.007 – these 17 genes are concurrently involved in catalytic (N = 12 proteins, p = 0.0047) or hydrolase (10 proteins, p = 0.0005) activities (GO functions), and that their functions are localized in extracellular regions (N = 14, p = 8.11e-7) or extracellular exosome (N = 12, p = 8.11e-7) and or lysosome (N = 6, p = 3.67e-5). Thus, the proteins involved in the detected degradation processes – typically occurring during cornification and bacterial invasions during estrus (i.e. a phase, which is characteristic of producing enucleated keratinized cells) – can be treated as markers of this particular phase. For example, Hornerin (HORN) is a component of the epidermal cornified cell envelopes^48^ and a marker of cell differentiation^49^. In our data, Hornerin was upregulated in estrus (3.9 fold) and significantly down-regulated in metestrus (3.4 fold). Hornerin is involved in the process of cell differentiation and with mucin/episialin (MUC1)^50^ forms anti-inflammatory epidermal barriers during estrus. The most differentially expressed protein in estrus (i.e. proestrus→estrus 5.5 fold, estrus→metestrus −5.1 fold) was Serpin A9 (SPA9). SPA9 is a member of the serpin family of serine protease inhibitors.

### Differentially expressed anti-microbial proteins

Our analysis (above) revealed that a biological process that is tightly linked to cell proliferation during the estrous cycle involves mechanisms of anti-microbial defence, because the total number of bacteria is highest during estrus and declines during metesturs^51^. Mucins, particularly MUC1, are effective barriers in the protection from microbial infection^50^. In our data, MUC1 was significantly up-regulated in estrus (4.5 fold). Similarly, MUC9 (i.e. OVGP1 - Oviduct-specific glycoprotein also known as mucin-9) was also up-regulated during estrus (4 fold) and metestrus. Another protein, which forms viscous barriers in oviducts under low pH during estrus, and which is up-regulated during estrus is Uromodulin (UROM, 2.3 fold). Significantly up-regulated anti-microbial proteins during estrus also involved alpha-macroglobulin or OVOS (Ovostatin, 3 fold) which is slightly down-regulated but not significantly during metestrus. Similar pattern is also typical for the Lysozyme g-like protein 1 (LYG1) which is up-regulated during estrus (2.5 fold) and down-regulated (4.8 fold) during metestrus. Other secretory antimicrobial proteins expressed in polarized epithelial cells involve those that bind to particular structures of bacterial membranes and those that physically break the membranes due to their amphipathic electrostatics. We have detected a member of the PLUNC family – the Lipopolysaccharide-binding protein (LBP, BPIFD2). The saliva proteome of the mouse contains seven members of the bactericidal/permeability-increasing proteins (i.e. BPI^52, 53^) which are male biased and include BPIA1, BPIB1, BPIB2, BPIB3, BPIFA2, BPIFB5, BPIFB9B^27^. BPI/PLUNC proteins have an antibacterial activity against gram-negative bacteria^52^. The vaginal fluid proteome, however, contains only LBP (BPIFD2), which is up-regulated during estrus (4.4 fold) and remains expressed during metestrus.

Anti-microbial proteins, which are significantly up-regulated during metestrus include the Cathelicidin antimicrobial peptide or CAMP/CRAMP (3 fold). A natural antibiotics CAMP forms an amphipathic alpha-helix similar to other antimicrobial peptides, and functional studies showed that CAMP is a potent antibiotics against gram-negative bacteria by inhibiting the growth of a variety of bacterial strains and is expressed by neutrophils and macrophages^54^. NGP (Neutrophilic granule protein) or ‘bectenecin’ – also belongs to cathelicidins, has a cathelicidin protein domain, and in our data, it is also significantly up-regulated in metestrus (2.8 fold), likely because it is co-expressed with CAMP in neutrophils which invade vaginal environment during metestrus. CAMP is regulated by the serine-proteases Kallikreins 5 and 7^55^ and thus belongs to an extended KLK/LEKTI network members that are crucial for homeostasis of stratified epithelia^56^. We did not detect KLK5 and KLK7 in the vaginal secretion. However, we have detected KLK1, KLK10, KLK11, KLK12, KLK13, and KLK14 invariantly expressed throughout the cycle, whilst KLK8 varied such that it was up-regulated during proestrus and estrus and down-regulated during metestrus (4.9 fold). SPINK5, which negatively regulates KLK5 expression was down-regulated (2.5 fold) during estrus.

### Lipocalins

Previously, it has been shown with microarrays that the mouse (CD 1) uterine transcriptome contains transcripts coding several major urinary proteins, namely *Mup3*, *Mup4*, *Mup5*, *Mup6*, *Mup9*, and *Mup20* ^57^. In their experiment, all detected *Mup* transcipts were significantly up-regulated in estrus (i.e. when compared to proestrus). Our data revealed a support for this trend on the level of protein. We have detected MUP20-darcin (unique peptides: VFVEYIHVLENSLALK, FAQLSEEHGIVR), MUP3, MUP5, and also a group of MUP proteins with no unique peptides. These peptides, however, are shared between the recently duplicated group-B MUPs and are, therefore, vizualized in Fig. 2B as the most likely hits though they are mixtures of several MUPs: sMUP17 (i.e. either MUP13 and/or MUP17), sMUP9 (i.e. MUPs 1, 2, 7, 8, 9, 10, 11, 12, 14, 15, 16, 18, 19). From the biological function perspective, it is plausible to group them simply for the fact that most group-B MUPs are highly homologous in *M*. *m*. *musculus*, have similar beta barrel structures, and there is almost no individual variability in the production of these proteins (i.e. at least in the liver/urine) in this sub-species of the house mouse^14–16^. Our proteomic data included also metestrus (i.e. when compared to citation^57^ where they concentrated on the proestrus/estrus analyses), thus we were interested whether MUPs and other lipocalins are predominantly expressed during estrus and declining in metestrus. A total of three MUPs were up-regulated in estrus: MUP20 (4.2 fold), the highly expressed sMUP9 (2.1 fold), and sMUP17 (3.2 fold) but all of them remained abundant in metestrus.

**Figure 3:**
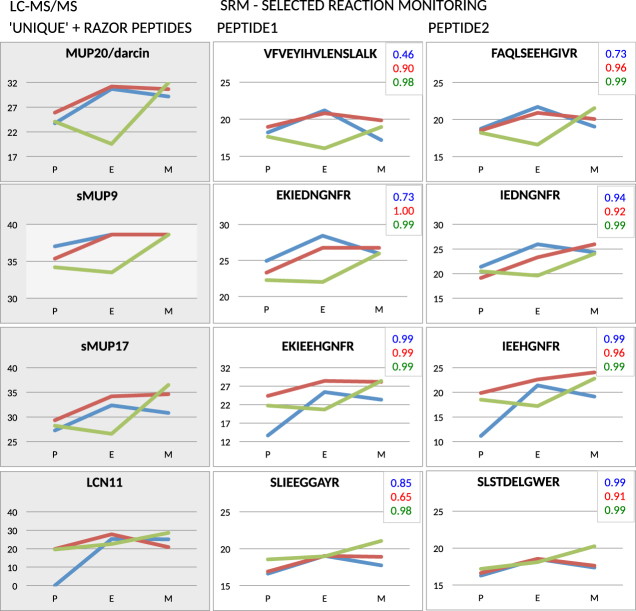
Selected Reaction Monitoring (SRM) of unique peptides for MUP20, sMUP9, sMUP17 and LCN11 using the triple quadrupole MS/MS instrument. We used this method as an alternative to Western blotting and revealed that untargeted LC-MS/MS data (first/grey column) are in a good agreement with targeted data from SRM (the second and the third columns). The level of agreement is supported by high correlation coefficients that are depicted in the inset of graphs with colours representing particular females. We have chosen two females representing the general trend from PLGEM modelling (females F7 – blue line, F8 – red line) and one female which up-regulated lipocalins in metestrus (F9 – green line). The x-axis labels represent proestrus (P), estrus (E), and metestrus (M).

To provide further evidence on the variation of significant MUPs and LCN11 (see below) and particularly MUP20-darcin, we have selected specific unique-peptide peaks and analysed their variation with Selected Reaction Monitoring (SRM) using triple quadrupole MS/MS instrument. This method is a MS-based equivalent to Western blotting but without antibodies^45^. We have chosen last three females of the dataset (F7, F7, F9) where two of them (F7, F8) followed the trend from PLGEM analysis whilst one female (F9) had a delay in the rising phase of protein abundance (F9 - green colour in Fig. 3). On the level of p < 0.05, the correlation between LC-MS and SRM data for particular females (differentiated by unique colours in Fig. 3) was high in all three females (note the insets with the correlation coefficients in Fig. 3). Thus, our SRM approach corroborated some of the trends detected with untargeted LC-MS/MS based approach.

For the first time (i.e. to our knowledge), we have detected the four members of the recently described^33^ family of odorant binding proteins OBP1 (KJ605390), OBP2 (KJ605391), OBP5 (KJ605392), and OBP7 (KJ605394) in vaginal fluids of the house mouse as highly abundant proteins. Our PLGEM analysis revealed significant up-regulations during estrus (e.g. OBP5 1.6 fold, p = 0.0027). However, the fold change is below 2 and it is in question whether these differences are biologically relevant. We have also detected lipocalins that have not been linked to chemical communication before. Lipocalin 11 (LCN11) (i.e. proestrus→estrus 2.3 fold) follows similar and significant trend as MUPs but its total expression is lower. Retinol-binding protein 1(RBP1) had an opposite trend (i.e. proestrus→estrus −2.13, estrus→metestrus 2.8 fold) with the lowest abundances during estrus. Siderocalin (LCN2) belons to a group of the most abundant proteins and was invariant throughout the cycle.

## Discussion

Vaginal fluids represent an important source of markers of reproductive states. Proteins obtained from the vaginal secretions by non-invasive lavage methods, and identified with the state-of-the-arts label-free proteomics enabled us to ask questions related to reproduction, chemical communication and evolution but with a detail that may help to support particular hypotheses dealing with the function of signalling proteins. The samples, however, are a mixture of proteins stemming out of multiple processes that take place in ovaries, uterine walls and horns, cervix, and various internal vaginal glands. In this paper, we aimed to characterize the expression pattern of proteins, which are known for their involvement in chemical communication with a particular focus on their variation throughout the estrous cycle of the wild derived house mice *Mus musculus musculus*. We used wild derived mice for the fact that the laboratory mice are influenced by the differential contribution of blocks of genes from the two house-mouse subspecies *M*. *m*. *domesticus* and *M*. *m*. *musculus* to current laboratory strains^58^ that may mask their natural behaviour.

We used the cytology screening of the cells from vaginal lavage and selected samples according to predominace of the cell types typical for each phase^1–3^. Consequent analyses of the vaginal fluid proteome served to corroborate our cytology determination. We have detected a total of 1.7% of proteins that are prevailingly expressed during estrus, Fig. 2B. They involve markers of cell keratinization as well as markers of catalytic activity and anti-microbial defence. Anti-microbial proteins function as natural innate-immune responses to an outburst of bacteria typically occurring during estrus^51^ such as Lysozyme G1 (LYG1) which has a hydrolase activity and is involved in the degradation of peptidoglycans from bacterial membranes (i.e. GO:0009253 peptidoglycan catabolic process). Bacterial peptidoglycans are continuously recognized by Peptidoglycan recognition protein 1 (PGRP1) which activates bacterial tool-component systems^59^ and is logically invariant throughout the cycle in our data.

A marker of interactions between symbiotic bacteria and the host is the highly expressed lipocalin 2/siderocalin (LCN2). In our data, the highly abundant LCN2 was invariantly expressed throughout the cycle, thus demonstrating that bacteria are present in all phases of the cycle. It is known that during metabolic degradation in most mucosal tissues, bacteria attempt to acquire ‘free’ iron by a secretion of high-affinity iron sequestrating siderophores. The mammalian host, however, limits this process by the production of Lipocalin 2^60^ which efficiently scavenges for catecholate-type siderophores^61^. Thus, high amounts of produced LCN2 represent an efficient regulatory element that prevents uncontrolled bacterial growth also in the mouse vaginal environment. WFDC proteins (i.e. ‘Whey acidic proteins four disulphide core’) were also shown to have anti-microbial properties^62^ and the two members WFDC12 and WFDC18 are present in the house mouse saliva as proteins encoded by the submandibular gland transcripts *Wfdc12* and *Wfdc18* ^27^. In this study, we have detected WFDC2 also invariantly expressed throughout the cycle. To add, an antimicrobial system of defence in vaginal environment, thus, seems to have two components, one that is static and invariant over the cycle, and another (dynamic) that reacts to bacterial growth and dynamically functions during estrus (e.g. LYG1, LBP, OVOS) and metestrus (e.g. CAMP, NGP).

Lipocalins of the laboratory mouse were already reported to vary between proestrus and estrus with the use of microarrays of the uterine horns^57^. They have provided evidence that all the detected lipocalin-coding transcripts, namely *Mup3*, *Mup4*, *Mup5*, *Mup6*, *Mup9*, and *Mup20* rise from proestrus to estrus. Our protein analysis corroborates their findings to some extent and extends their view because we included also metestrus. We show for the first time in wild-derived *M*. *m*. *musculus* mice that, MUP20 or ‘darcin’ is the most up-regulated lipocalin when passing from proestrus to estrus. This is interesting because some studies originally thought that urinary MUP20 is a male unique lipocalin with pheromonal effects even without ligands and which stimulates an inherent attraction for particular males^63^. Here we provide evidence, that MUP20 is not male-unique because it is produced by females in their vaginal secretions during estrous cycle (i.e. *M*. *m*. *musculus*) and also by females of the laboratory mice in their uterine horns^57^. Although, we have no data on what MUP20 binds in the vaginal secretions, we show that MUP20 production significantly rises and reaches its peak in estrus. Interestingly, major urinary proteins were also detected in metestrus which – if they were supposed to signal receptivity – would decrease a male’s chances to correctly evaluate the best time for mating. Furthermore, although male-biased, MUP20 and other MUPs are also produced by salivary^27^ and lacrimal^26^ glands of males and females. It is also possible that MUP20 plays different roles in different tissues which may be further explored with behavioural experiments and/or with GC-MS techniques.

There is as yet another alternative explanation to why are some lipocalins expressed also during metestrus. It is possible that MUPs play other roles besides sexual signalling similarly as olfactory receptors play other roles besides the detection of chemical signals^64^. We already suggested this alternative to a common view that MUPs serve only to chemical communication by providing evidence that MUPs are expressed in a sexually dimorphic manner in one tissue (e.g. liver,^13^) but are non-dimorphic and with lower expression levels elsewhere (e.g. saliva^27^ and tears^26^). Here we suggest a likely hypothesis that lipocalins (i.e. including major urinary proteins and odorant binding proteins) may have the potential to detoxify mucosal tissues by removing various organic compounds. We have postulated this explanation as the ‘Toxic waste hypothesis’^19^ which states that the same products of metabolic degradation might have been an ideal source of signals that have driven the evolution of chemical communication by means of a consequent toxic-waste perception and recognition. Some of these organic compounds might have come from bacterial degradation because we found various antimicrobial proteins differentially acting during estrous cycle. This hypothesis, however, needs to be further tested with GC-MS techniques. Furthermore, lipocalin involvement in detoxification has been experimentally evidenced, so for example MUPs are already known to transport toxic substances (i.e. other than known pheromones) out of the body^65^.

To conclude, MUPs and OBPs may function as the transporting devices of volatiles to signal female receptivity in the house mouse, *M*. *m*. *musculus*. The most abundant lipocalins during estrus were MUP20, and the group-B MUPs - sMUP9 and sMUP17. However, they remain expressed during metestrus. Thus, it is likely that these proteins may have other roles besides sexual signalling. We have also detected variation in bactericidal proteins, thus further supporting an idea that females may have adopted alternative strategies in controlling microbiota thus yielding different odor profiles throughout the cycle.

## Electronic supplementary material

Dataset 1
