# Supplementary material for: Differential regulation of vaginal lipocalins (OBP, MUP) during the estrous cycle of the house mouse
Source: Sci Rep. 2017 Sep 15;7:11674. doi: 10.1038/s41598-017-12021-2 (PMC5601457; doi:10.1038/s41598-017-12021-2)
Supplement: Supplementary file 1 — Dataset 1 [file 41598_2017_12021_MOESM1_ESM.pdf]

Differential regulation of vaginal lipocalins (OBP, MUP) during the estrous cycle of the house mouse

Martina Černá[1], Barbora Kuntová[1], Pavel Talacko[1], Romana Stopková[1], & Pavel Stopka[1,*]

[1] *BIOCEV group, Department of Zoology, Faculty of Science, Charles University, Viničná 7, CZ 12844, Czech Republic*

[*] Correspondence: Pavel Stopka pstopka@natur.cuni.cz

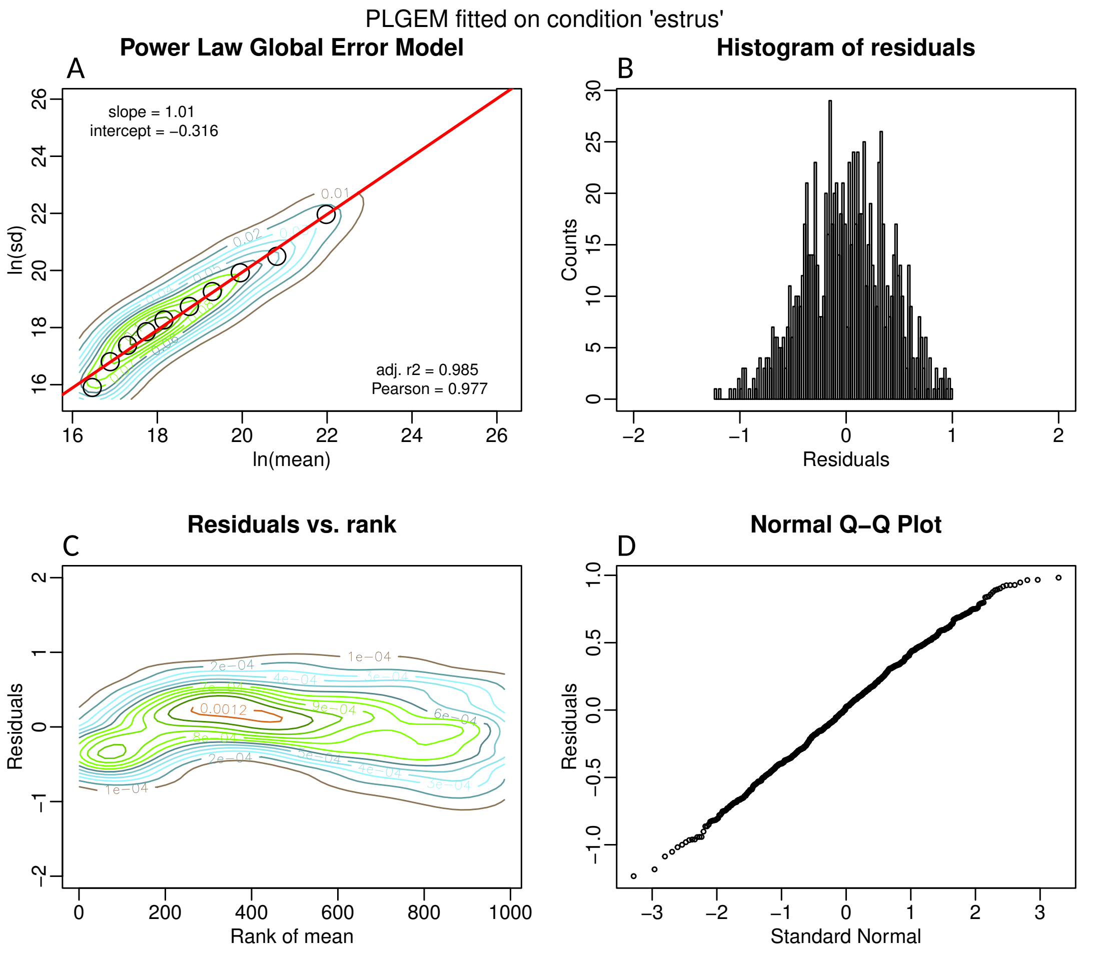

Supplementary figure 1: Details of the Power Law Global Error Model (PLGEM) [37]: A – the model fitting on a female experimental condition (i.e. estrus) thus setting the baseline for PLGEM modelling, B – the histogram of residuals shows symmetric data distribution over the x=0, C – distribution of residuals show a flat distribution over the y=0, and D – the normal quanitle-quantile plot shows almost the straight line (y=x) thus revealing that PLGEM is an amenable model to test differences in protein abundances from our normalized data (see citation [37]).
